# Painless Giant Submandibular Gland Sialolith: A Case Report

**DOI:** 10.7759/cureus.19429

**Published:** 2021-11-10

**Authors:** How Kit Thong, Iylia Ajmal Othman, Roszalina Ramli, Primuharsa Putra Sabir Husin Athar

**Affiliations:** 1 Otolaryngology - Head and Neck Surgery, KPJ Seremban Specialist Hospital & KPJ Healthcare University College, Seremban, MYS; 2 Otolaryngology - Head and Neck Surgery, International Islamic University Malaysia Medical Centre, Kuantan, MYS; 3 Oral and Maxillofacial Surgery, Faculty of Dentistry, Universiti Kebangsaan Malaysia Medical Centre, Kuala lumpur, MYS

**Keywords:** wharton's duct, submandibular gland excision, submandibular gland, sialolithiasis, giant sialolith

## Abstract

Sialolithiasis is one of the most common diseases involving the salivary glands. It is a condition that occurs due to an obstruction in a salivary gland or its duct due to a calculus. The formation of a salivary stone is believed to be secondary to the deposition of mineral salts around a nidus, which is frequently associated with a recurrent bacterial infection. Patients with submandibular sialolithiasis usually present with acute swelling over the neck associated with pain, fever, and purulent intraoral discharge. The size of the calculus varies from <1 mm to a few centimeters. The frequency of sialolithiasis is relatively common. It is estimated to affect 12 in 1000 of the adult population. However, the occurrence of giant sialoliths, >15 mm in any diameter, is rare. Here, we describe our experience with a case of giant submandibular sialolithiasis measuring 25 mm presenting as a painless submandibular mass. The patient underwent submandibular gland excision followed by a full recovery.

## Introduction

Sialoliths are the most common diseases of the salivary glands after mumps. This condition affects 0.1%-1.0% of the adult population, typically between the ages of 30 and 60 years, with a higher incidence in males [1]. Only small percentages of sialolithiasis cases have been reported in the pediatric population to date [1,2]. Most sialoliths develop in the submandibular gland, with the duct being frequently more affected than the parenchyma, followed by the parotid gland, and small percentages affecting the sublingual and other minor glands [2,3].

Salivary calculi generally consist of an amorphous mineralized nucleus, surrounded by concentric laminated layers of organic and inorganic substances. The organic components of salivary stones include collagen, glycoproteins, amino acids, and carbohydrates. The major inorganic components are hydroxyapatite, carbonate apatite, whitlockite, and brushite [4]. The etiological factors involved in the formation of sialoliths can be divided into two groups: the first group describes salivary retention secondary to morpho-anatomic factors as the contributing factors (seen in cases of salivary duct stenosis, salivary duct diverticulum, etc.), while the other group primarily describes increased viscosity of saliva as the etiological factor (high supersaturation, crystallization inhibitor deficit, etc.) [1].

Commonly, a sialolith measures from 1 to less than 10 mm, with a mean size reported as 6-9 mm. Giant sialoliths are rare and classified as those measuring >15 mm in one dimension [5].

The aim of this paper is to present a case of an unusually sized sialolith and a literature review of current thoughts on preferred technique for imaging the salivary gland, as well as various treatment modalities.

## Case presentation

A 71-year-old male presented with a two-week history of painless right submandibular swelling that was not associated with fever. The patient had underlying hypertension and diabetes mellitus that were regularly treated.

**Figure 1:**
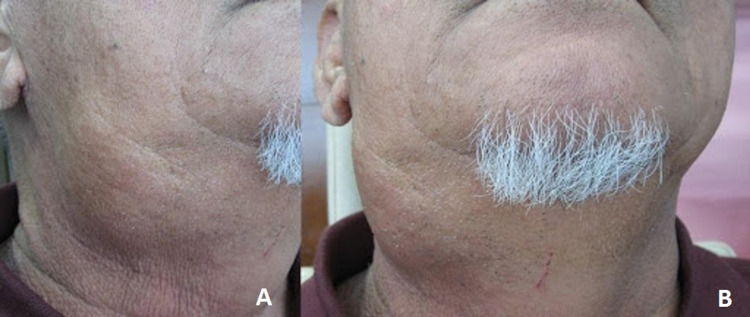
Right submandibular swelling (A) Lateral view of the right neck with an obvious submandibular swelling. (B) Anterior view of the neck with the same swelling extending inferiorly to the level of the thyroid cartilage.

On examination, a right submandibular swelling with normal overlying skin measuring 6 × 5 cm that was non-tender, mobile, and firm in consistency was noted (Figure 1A, 1B). The swelling is ballotable by bimanual palpation. There was no other swelling palpable in the neck region. Intraorally, pus was noted at the Wharton’s duct orifice, and no sialolith was palpable.

**Figure 2:**
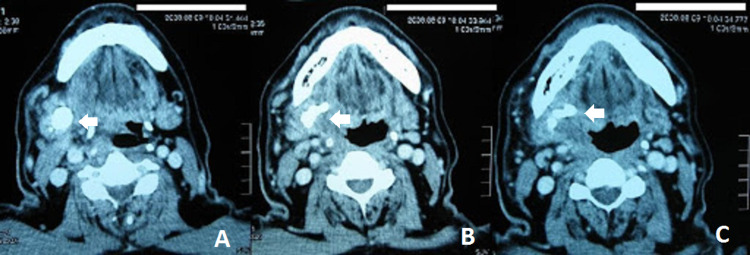
Axial images of the preoperative CT scan of the neck showing an opacity (white arrows) in the right submandibular gland and duct (A) A round opacity in the parenchyma of the right submandibular gland. (B,C) Medial extension of the sialolith to the proximal submandibular duct.

**Figure 3:**
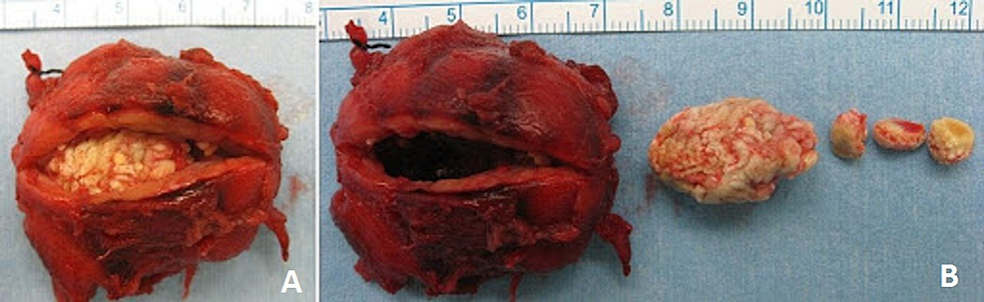
Position of the stone in the gland parenchyma with smaller stone fragments in the proximal submandibular duct (A) A large stone within the submandibular gland parenchyma. (B) The arrangement of the stone with its extension into the proximal submandibular duct.

Preoperative blood investigations (complete blood count, serum urea and electrolytes, and serum uric acid), electrocardiography, and chest radiographs were normal. Computed tomography (CT) of the neck was performed as part of the preoperative assessment, which showed opacity in the right submandibular gland and duct (Figure 2A, 2B, 2C). A diagnosis of right submandibular stone was made. The patient subsequently underwent excision of the right submandibular gland under general anesthesia. Intraoperatively, the right submandibular gland was indurated (Figure 3A). During the excision, the surgeon noted another firm bulge along the submandibular duct that turned out to be a few smaller pieces of stones within the duct (Figure 3B). The size of the largest stone was 25 mm. Postoperative recovery was uneventful. Histopathology examination revealed severe acute-on-chronic sialadenitis with multiple calculi.

## Discussion

Commonly, a sialolith measures from 1 to less than 10 mm, with a mean size reported as 6-9 mm. Giant sialoliths are rare and classified as those measuring >15 mm in one dimension [5]. In our case, the size of the main sialolith was 25 mm and completely encased in the glandular substance with part of it located in the proximal aspect of the submandibular duct. Removal of the affected submandibular gland was performed, considering the size, location, and involvement of the proximal submandibular duct.

The diagnosis of sialolithiasis can be made on the basis of its clinical presentation and careful examination. It often presents as recurrent bouts of pain and swelling of the affected salivary gland, usually during mealtimes or in response to other salivary stimuli. In this case, the stone reached its current size without any pain. Bimanual palpation of the floor of the mouth may reveal a palpable stone in most cases. A uniformly firm and hard gland also suggests a hypofunctional or nonfunctional gland [3].

Radiological investigations are deemed very useful in diagnosing sialolithiasis. Radiopaque stones (more than 80% of cases) can be visualized on standard mandibular occlusal radiographs. Radiolucent stones or stones deep within the glands can be investigated using sialography. However, sialography is contraindicated in acute sialadenitis or patients who are allergic to contrast. Ultrasonography (USG) with a high-frequency linear transducer (10-13 MHz) is considered the gold standard and first investigation of choice for parenchymal stones. Its sensitivity, however, decreases in ductal and papillary diseases. Andretta et al. proposed sialo-magnetic resonance imaging (sialo-MRI) as a noninvasive tool used for diagnosing sialolithiasis [6]. Sialo-MRI produces sialographic images without using contrast medium injection and without the disadvantages of ionizing radiation (as used in CT and contrast sialography). Sialo-MRI is superior to USG because the gland’s anatomic structure remains unchanged, allowing exact visualization of its ducts and acini, compared with USG, which is operator-dependent, and the anatomic structure may be compressed by the transducer. This technique also allows functional evaluation of the parenchyma and glands after citric acid stimulation. Acute and chronic inflammation differs in their signal intensities, particularly on T2-weighted images. Sialo-MRI, however, is used as an adjunct to plain radiographs or USG as it cannot directly visualize the presence of a stone. Indirectly, sialo-MRI shows proximal salivary duct dilation in the presence of a stone [6].

The role of CT has not been discussed much. As this modality is readily available and the procedure is not time-consuming, it has become a standard investigation tool in our center. The locality of the stones can be determined (intraductal or intraparenchymal), as well as the size and number of stones present. These findings will facilitate the surgeon to decide on the best operative method for removal. Interventional sialendoscopy can be used as both a diagnostic and treatment tool [7,8]. However, the procedure should be performed under the supervision of experienced surgeons who have undergone specialized training, as it can be technically challenging. With the aid of CT preoperatively, surgeons can gauge the size of the stones (if suitable for endoscopic removal) and the location of the stones, especially if there appears to be more than one at different sites.

Essentially, the treatment goal is to restore normal salivary gland function. Treatment options may be selected based on the clinical presentation, size, and site of the obstructing stone. Small stones can be “milked out” through ductal orifices by means of bimanual palpation with warm heat application and sialagogue administration. Most cases of sialolithiasis respond to antibiotics with simple sialolithotomy [9]. Large stones or those located in the proximal duct may warrant piezoelectric extracorporeal shock wave lithotripsy prior to surgical removal [6]. In some cases, excision of the whole gland may be deemed necessary, as shown in this case.

## Conclusions

Our case report describes the removal of a giant sialolith that required excision of the submandibular gland. There are various methods for the treatment of sialolithiasis depending on the affected gland and the size and site of the stones. However, whenever possible, one should choose the most conservative method or the treatment most suitable to the specific situation of the patient.
